# Efficacy of a Mandibular Advancement Appliance on Sleep Disordered Breathing in Children: A Study Protocol of a Crossover Randomized Controlled Trial

**DOI:** 10.3389/fphys.2016.00353

**Published:** 2016-08-19

**Authors:** Ghassan Idris, Barbara Galland, Christopher J. Robertson, Mauro Farella

**Affiliations:** ^1^Craniofacial Biology and Clinical Oral Physiology Research Programme, Sir John Walsh Research Institute, University of OtagoDunedin, New Zealand; ^2^Department of Women's and Children's Health, University of OtagoDunedin, New Zealand

**Keywords:** behavior, growth hormone, mandibular advancement appliance, quality of life, sleep apnea, sleep disordered breathing, snoring

## Abstract

**Background:** Sleep-Disordered Breathing (SDB) varies from habitual snoring to partial or complete obstruction of the upper airway and can be found in up to 10% of children. SDB can significantly affect children's wellbeing, as it can cause growth disorders, educational and behavioral problems, and even life-threatening conditions, such as cardiorespiratory failure. Adenotonsillectomy represents the primary treatment for pediatric SDB where adeno-tonsillar hypertrophy is indicated. For those with craniofacial anomalies, or for whom adenotonsillectomy or other treatment modalities have failed, or surgery is contra-indicated, mandibular advancement splints (MAS) may represent a viable treatment option. Whilst the efficacy of these appliances has been consistently demonstrated in adults, there is little information about their effectiveness in children.

**Aims:** To determine the efficacy of mandibular advancement appliances for the management of SDB and related health problems in children.

**Methods/design:** The study will be designed as a single-blind crossover randomized controlled trial with administration of both an “Active MAS” (Twin-block) and a “Sham MAS.” Eligible participants will be children aged 8–12 years whose parents report they snore ≥3 nights per week. Sixteen children will enter the full study after confirming other inclusion criteria, particularly Skeletal class I or class II confirmed by lateral cephalometric radiograph. Each child will be randomly assigned to either a treatment sequence starting with the Active or the Sham MAS. Participants will wear the appliances for 3 weeks separated by a 2-week washout period. For each participant, home-based polysomnographic data will be collected four times; once before and once after each treatment period. The Apnea Hypopnea Index (AHI) will represent the main outcome variable. Secondary outcomes will include, snoring frequency, masseter muscle activity, sleep symptoms, quality of life, daytime sleepiness, children behavior, and nocturnal enuresis. In addition, blood samples will be collected to assess growth hormone changes.

**Trial registration:** This study was registered in the Australian New Zealand Clinical Trials Registry (ANZCTR): [ACTRN12614001013651].

## Introduction

The health impact of sleep-disordered breathing (SDB), particularly, obstructive sleep apnea (OSA) has been increasingly recognized in both adults and children (Marcus, 2001; Casale et al., 2009). The prevalence of OSA in children varies from 1 to 4% (Rosen et al., 2003; Bixler et al., 2009; Bonuck et al., 2011), while most authors report a 10% prevalence of habitual snoring in children (Lumeng and Chervin, 2008). SDB has been associated with growth disorders (Marcus et al., 1994), daytime sleepiness (Li and Lee, 2009), educational and behavioral problems (Blunden et al., 2000; Galland et al., 2015), and nocturnal enuresis (Stone et al., 2008). In the most severe cases, OSA may have life-threatening consequences like cardiorespiratory failure, which can lead to death (Ross et al., 1987; Lavie, 2004).

Enlarged adenoids and tonsils are the most common etiology of OSA in children. Obesity during childhood represents another important risk factor for OSA (Horner et al., 1989; Gozal and Kheirandish-Gozal, 2009). Craniofacial anomalies are also associated with changes in airway morphology and respiratory problems (McNamara, 1981). These anomalies, basically, include maxillary and mandibular retrognathia, increased facial height, decreased facial width, increased overjet, and palatal crossbite (Lofstrand-Tidestrom et al., 1999; Cakirer et al., 2001; Kawashima et al., 2002; Li et al., 2002; Ozdemir et al., 2004; Juliano et al., 2009; Pirila-Parkkinen et al., 2009; Flores-Mir et al., 2013; Katyal et al., 2013).

Polysomnography (PSG) is considered the gold standard for SDB diagnosis (Casale et al., 2009), and the apnea hypopnea index (AHI) is the main outcome to aid diagnosis. AHI is defined as the number of apnea and hypopnea events recorded per hour of sleep. A pediatric OSA event, relying on PSG, is defined as absence of airflow for two respiratory cycles (2 breaths) with continued chest wall and abdominal wall movement (Berry et al., 2012). Whereas, obstructive hypopnea is defined as a decrease in nasal flow ≥30% (lasting at least 2 breaths) with a corresponding decrease in oxygen saturation ≥3% and/or arousal (Berry et al., 2012).

Full PSG is costly to perform in sleep laboratory, and scoring sleep stages is time consuming (Collop et al., 2007). Consequently, over the last decades, there has been increased interest in cost-effective methods of diagnosing SDB within the home setting using portable monitoring devices (Bridevaux et al., 2007; Polese et al., 2010). Type III home PSG, also referred to as abbreviated PSG without electonecephalogram (EEG), electrooculogram (EOG), and electromyogram (EMG), has been used in the majority of the published studies on home testing of SDB in children; this abbreviated PSG, is more suitable for use in children, it is less time-consuming, and it is easier to set up and score (Tan et al., 2015).

The first line and the most common treatment for pediatric OSA is adenotonsillectomy (Li and Lee, 2009; Marcus et al., 2012). Although significant improvements in the AHI are observed following adenotonsillectomy, full resolution of OSA (AHI ≤ 1) occurs only in 27–60% of cases (Friedman et al., 2009; Bhattacharjee et al., 2010). Adenotonsillectomy is also associated with risk of postoperative complications beyond the anesthetic and surgical procedures. For example, OSA children show a significantly higher risk of respiratory compromise, defined as intermittent or continuous oxygen saturation of 70% or less, and/or hypercapnia, requiring intervention after surgery (McColley et al., 1992; Gross et al., 2006). Children with obesity, severe OSA, or craniofacial abnormalities may require more protracted inpatient care and/or intensive care unit observation (Leong and Davis, 2007; Isaacson, 2014). Studies have reported an accelerated mandibular ramus growth and improved facial morphology after adenotonsillectomy in pediatric OSA patients (Agren et al., 1998). This improvement may be due to normalization in growth hormone (GH) serum levels, also to a change of tongue and cheek posture (Woodside et al., 1991). However, this growth acceleration is not sufficient to correct the malocclusion and the underlying skeletal discrepancy often requires subsequent dentofacial growth modification treatment (Peltomäki, 2007).

Continuous positive air pressure (CPAP) is the primary treatment option for adult OSA (Casale et al., 2009). CPAP is also advocated for severe pediatric SDB (Agren et al., 1998), or for persistent OSA after adenotonsillectomy, but it is generally not well tolerated by children (Marcus et al., 2012). Adherence to CPAP treatment in children is a major problem, with rates of only 30–70% (O'Donnell et al., 2006; Uong et al., 2007). From a biomechanical stand-point, there are anecdotal reports of potential facial growth disturbances from long-term use of CPAP due to the elastic strap which maintains the mask, as it may apply a restraining force on the growing maxilla (Conley, 2011).

Oral appliances have also been widely used for the treatment of OSA, particularly in adults (Villa et al., 2002; Ferguson et al., 2006; Casale et al., 2009). These appliances increase the posterior oropharyngeal airway and reduce upper airway collapsibility by holding the mandible in a protruded position during sleep; furthermore, the device may trigger stretch receptors, which in turn activate the airway supporting muscles (Casale et al., 2009). Previous studies have shown that oral appliances are better tolerated than CPAP treatment (Ferguson et al., 2006) because of improved comfort, quietness, and portability. Mandibular advancement appliances are the most common type of oral appliances used in the treatment of SDB in adults, but their use in children is less common, with little information about their efficacy (Ferguson et al., 2006). The few studies on the subject suffer from important methodological flaws, making it difficult to reach a definitive conclusion about the efficacy of MAS appliances in children with SDB (Villa et al., 2002; Cozza et al., 2004; Carvalho et al., 2007; Huynh et al., 2015; Nazarali et al., 2015).

## Objectives

Aims of the study are:(1) to test the short-term efficacy of MAS in the treatment of children with SDB; and (2) to assess the effect of MAS treatment on quality of life, behavior, growth hormone levels, and nocturnal enuresis in SDB children.

## Materials and methods

### Study design

This study will be carried out as a single-blind crossover randomized controlled trial. Each participant will wear two appliances consisting of an active MAS and non-active MAS (Sham MAS). Each appliance will be worn for a 3-week treatment period, which will be separated by a 2-week washout period. A similar study design and treatment time have been used in previous studies comparing the efficacy of MAS to nasal CPAP treatment in adults (Ferguson et al., 1996, 1997).

This study will be conducted in the Discipline of Orthodontics, School of Dentistry University of Otago, New Zealand. Ethical approval has been granted by the Human Ethics Committee at University of Otago [H14/054], and New Zealand Health Research South Ethics Committee- Southern District Health Board [01050]. The research has also been approved by Ngāi Tahu Research Consultation Committee (Research consultation with Māori).

### Participants

#### Sample size estimation

A reduction of 50% in AHI is generally considered clinically relevant (Ferguson et al., 2006). This change corresponds to a large effect size (Cohen's *d* = 1.5). To detect this effect size, and setting α error to 0.05 and β error to 0.20 (one-tail test) using a repeated-measurement design, we have estimated that at least 13 participants are needed. We will recruit 16 children to account for possible dropouts.

#### Recruitment

We will advertise in local newspapers to invite children who live in Dunedin city/New Zealand, to participate in the study according to the following eligibility criteria: age 8–12 years; parental report of snoring three nights or more per week.

Eligible patients should meet the following selection criteria:

A score for the Pediatric Sleep Questionnaire (PSQ) ≥0.33 (Chervin et al., 2000); class II or class I incisor relationships; Skeletal maxillomandibular relations of class I or class II which will be confirmed by lateral cephalometric radiograph (ANB angle and Wit's appraisal; Steiner, 1959; Jacobson, 1975).
Exclusion criteria include severe OSA defined as an AHI >10 (Association ASA, 2013); craniofacial syndromes and genetic syndromes; neuromuscular diseases; or Angle class III incisor relationships; previous orthodontic treatment.
Patients diagnosed with severe OSA will be referred to a Medical Specialist.

#### Oral appliances

**Figure 1:**
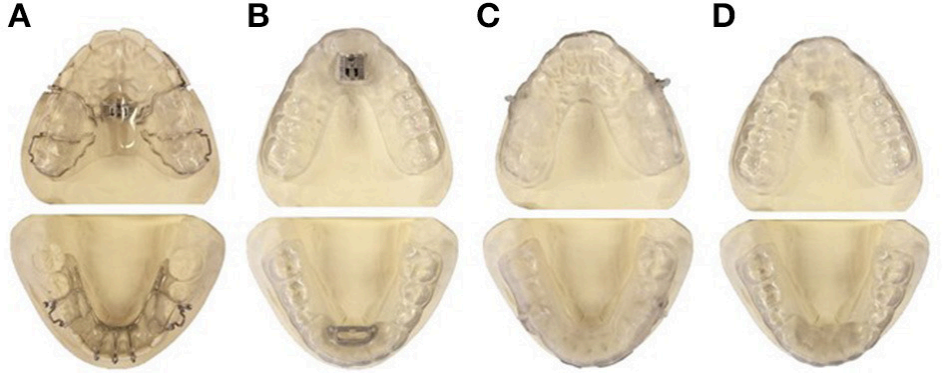
**Appliances tested in a pilot study to identify the most suitable appliance design to be used in the main study. (A)** Traditional Twin-block: Removable upper and lower acrylic plates, each plate has matching pieces to encourage the lower jaw to slide forward, bilateral hooks were added to insert vertical elastics. **(B)** Metallic fastener Twin-block: Removable upper and lower vacuum formed plates and a metallic fastener (TAP) is used to hook the upper and the lower plates enabling the mandible to be advanced forward. **(C)** Clear elastic Twin-block: Removable upper and lower vacuum formed plates with matching pieces to encourage the lower jaw to slide forward, bilateral plastic hooks were added to accommodate vertical elastics. **(D)** Sham Twin-block: Removable upper and lower vacuum formed plates, without any lower jaw advancing device.

An n-of-1 pilot study was undertaken to compare the acceptance, and the mechanical retention of different splint designs to choose the best design to be used in the main study. Four appliances were identified and tested in an 11-year old boy with class II dental and skeletal jaw relationships, the appliances were: Traditional Twin-block (Clark, 1988); Twin-block with metallic fastener (TAP®-splint, SCHEU Dental Technology, Brussels, Belgium); Clear elastic Twin-block; Sham Twin-block (Figure 1).

As discomfort affects adaptation and acceptance (Sergl and Zentner, 1998; Idris et al., 2012), this was evaluated in a pilot study using two assessment methods: the participant was firstly asked to report how comfortable was each appliance immediately after insertion into the oral cavity in compare to the other appliances. The four appliances were presented in pairs to compare each appliance to the remaining three (a total of 12 pairs were generated to avoid confounding caused by sequence of appliances application); the participant was instructed to insert an appliance into the mouth and he was asked to move the mandible to the right and left, front and back, and then to swallow, then to repeat the same to another appliance; after that the participant was asked to indicate which of the two appliances was more comfortable to wear. The second method aimed to assess acceptance after wearing the appliance for 2 h while awake and during one night sleep. One night appliance-free was allowed as washout period between two sequentially-tested appliances. Discomfort was assessed by using a 100-mm visual analog scale (VAS) with words (worse than I can imagine; better than I can imagine) anchored at the left and right end of the scale, corresponding to worse discomfort and best comfort, respectively. The participant rated the discomfort intensity by putting a vertical slash on the line that best represented the intensity of discomfort. The higher the VAS score the better the appliance acceptance. Two separate VAS scores were collected after both the awake and asleep wearing periods.

Retention was tested using both a subjective and an objective assessment. Subjective assessment was made by asking the participant (1) if the appliance was held in the mouth the whole night, (2) if the upper and lower splints were fitting well to the teeth, and (3) if the upper and lower splints stay attached to each other. Response options were yes/no.

Passive and active retention were also objectively tested by two independent examiners (GI, MF) by checking whether the appliance was stable in-mouth upon, (1) maximum jaw opening and, (2) direct pulling of the examiner.

Since the traditional Twin-block appliance showed the best retention and acceptance this was chosen as the study protocol intervention. Additional arguments in support of this decision were:

- It is easy to titrate and can also be used to expand the upper arch.
- It includes metal clasps to increase retention as required.
- It is very popular amongst orthodontists and has been extensively tested in previous research.
- It has a low failure rate and is well tolerated by children (Morris et al., 1998).
- It can be used for facial growth modification in patients with skeletal class II jaw relationship.

#### Twin-block design (active MAS)

**Figure 2:**
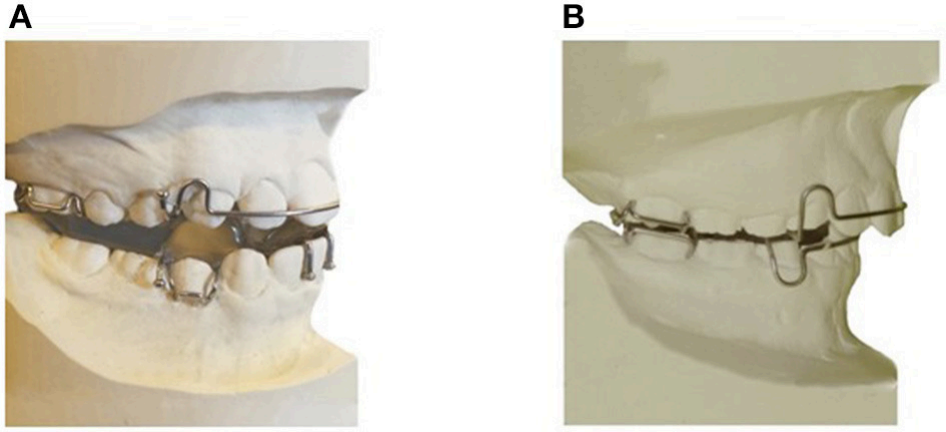
**Appliances to be used in the study. (A)** Twin-block appliance (active MAS). **(B)** Sham MAS (non-active MAS).

This consists of two removable upper and lower plates; each plate has matching pieces which encourage the lower jaw to posture or slide forward as the teeth come together (Figure 2) (Clark, 1988). Hooks will be added to the Twin-block bilaterally to insert vertical elastics. These elastics will hold the mouth closed during sleep and the mandible postured in a forward position (Barnes et al., 2004). The construction bite of the appliance will be determined using the George Gauge™(George, 1992), which allows an accurate determination and registration of the mandibular advancement. The bite registration will be taken for all participants by advancing the mandible 75% of the maximum protruded position of the jaw with minimum bite opening.

#### Sham MAS design (non-active MAS)

This consists of two non-active upper and lower acrylic plates resembling the design of the active MAS, but without any component to protrude the mandible (Figure 2).

### Randomization

Participants will be randomly assigned to either a sequence starting with the Twin-block or the Sham MAS. Thereafter, participants will wear the appliances for 3 weeks separated by a 2-week washout period. Participants will not be told whether they wear the active or the non-active appliance. Each night of wear time will be recorded by parents using diaries.

**Figure 3:**
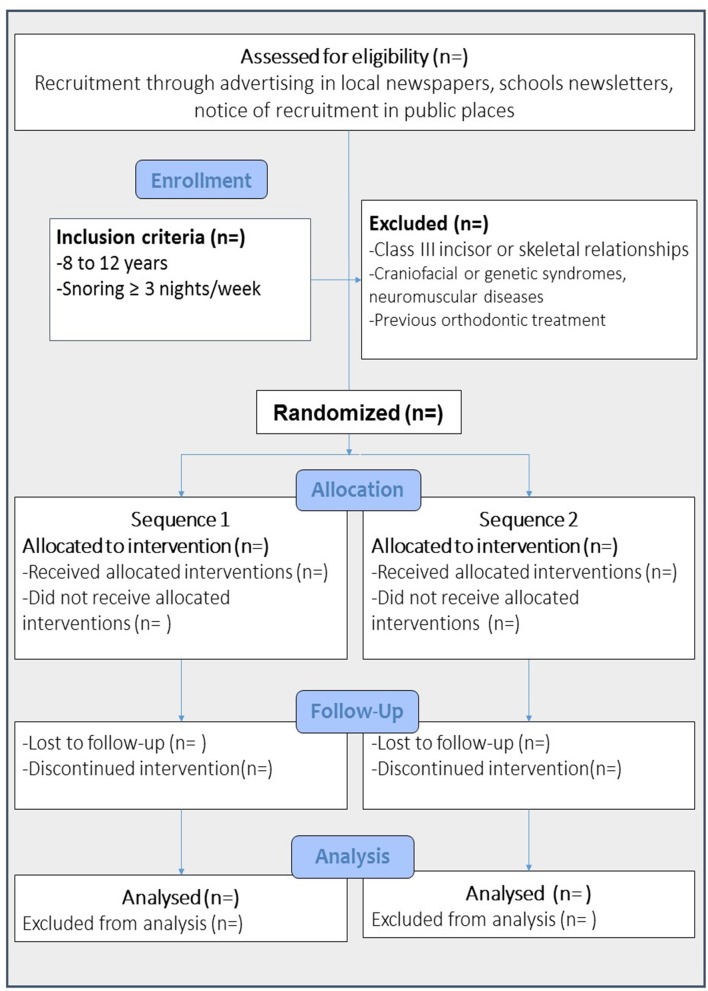
**Participants' flow in the study**.

Patients will be allocated to active or sham treatment using four randomized n-of-4 blocks that are balanced for treatment sequence (Sequence 1: Twin-block MAS first followed by Sham MAS; sequence 2: Sham MAS first followed by Twin-block MAS; Figure 3). Allocation will be concealed in opaque envelopes, and disclosed by a member of the research team (MF) immediately after enrolment. Participant flow is illustrated in (Figure 3).

## Outcome measurements and stepwise procedure

### Sleep data

Home-based abbreviated polysomnography, Type III (Polese et al., 2010), will be performed using a portable monitoring unit (Embletta MRP PG-XS-ENU, Natus Neurology Incorporated, Ontario, Canada).

PSG recording channels in the Embletta unit will be comprised of one electromyographic (EMG) channel, a finger pulse oximetry, nasal airflow (using a nasal pressure catheter), nasal and mouth thermistry, thoracic and abdominal respiratory effort bands (two piezoelectric belts will be placed around the rib cage and abdomen and monitor respiratory movement), body position sensors, and a microphone attached to the nasal cannula extension. The following variables will be measured: pulse oximetry, airflow, respiratory effort, snoring sound, body position, and EMG of the masseter muscle. This equipment has previously been validated for the diagnosis and management of SDB (Dingli et al., 2003; Skomro et al., 2010; Fredheim et al., 2014; Park do et al., 2015).

The portable device is set up through the use of a separate application software, RemLogic-E version 3.4 (Embla system, Natus Europe, Planegg, Germany). Channels are configured and identified, sampling rates set, filtering options defined, recording timing (on/off times), etc. through the same software.

**Figure 4:**
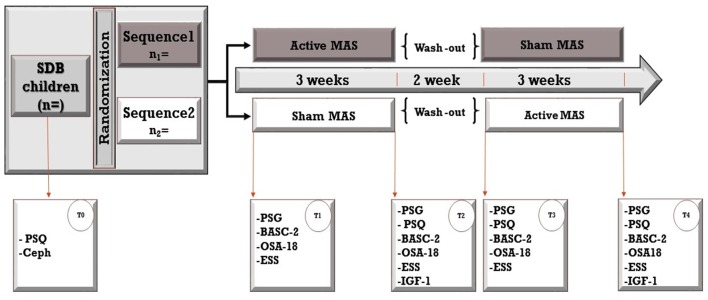
**The study design is a crossover randomized controlled trial**. Sixteen patients will be randomly assigned to two sequences; both sequences include a 3-week treatment period with active and non-active (sham) mandibular advancement splints but in a different order (Active followed by Non-active and Non-active followed by Active). Treatment periods will be separated by a 2-week washout period. Assessments will be taken at baseline (T0) and four times throughout the study. PSF, polysomnography; Ceph, Cephalograms; PSQ, Pediatric Sleep Questionnaire; BASC-2, Behavior Assessment System for Children, second edition; OSA-18, Quality of Life questionnaire; ESS, Epworth Sleepiness Scale (ESS).

Home-based PSG data will be collected four times for each participant at baseline and after treatment with Twin-block or Sham MAS, with the support of a research assistant (Figure 4).

The AHI will be calculated according to the American Academy of Sleep Medicine (AASM) criteria for scoring pediatric respiratory sleep studies (Berry et al., 2012). AHI is the main outcome of interest and sleep position will be considered in AHI scoring because sleep related obstructive respiratory events in children occur more commonly in the supine sleep position (El-Kersh et al., 2016).

### Snoring frequency

Snoring sounds will be assessed by analyzing audio recordings recorded using the portable PSG equipment. Snoring will be scored manually by listening to the audio recording using RemLogic-E software and the result will be reported as: total snoring time, relative snoring time, number of snoring episodes, average snoring episodes, and longest snoring episode.

The use of audio recordings is one of the recommended methods to aid visualize snoring oscillations (Berry et al., 2012). Embletta MPR microphone (an analog omnidirectional condenser microphone) attached to nasal cannula will record the snoring sounds. All signals will be fed into analog/digital (A/D) converter with a 200 Hz sampling rate (each channel has 200 points or samples per second; Shiomi et al., 2011). For optimal visualization of snoring, a high-pass filter to remove very low frequencies (20 Hz) and a low-pass filter to remove high frequencies (3 kHz) will be used, this will permit acquiring relevant information of snoring sounds (Pevernagie et al., 2010).

Subjective reports of snoring frequency will also be collected by parents using daily diaries throughout each treatment period (Figure 4).

### Growth hormone levels

Blood samples will be taken from all participants by a certified phlebotomist in a medical laboratory (Southern Community Laboratories). Growth hormone will be indirectly assessed by determination of insulin-like growth factor-1 (IGF-1) levels (Laron, 2001).

Two samples will be collected at the end of each treatment period and collected in a non-fasting state in the morning to early afternoon.IGF-1 concentrations were assayed using a human IGF-I quantikine ELISA Kit (PDG100, R&D Systems, Minneapolis, USA). The human IGF-1 immunoassay employs the quantitative sandwich enzyme technique. Samples will be stored in a −80°C freezer until batch assayed by a technician who will be blinded to the treatment period. Disposal of medical wastes will follow the World Health Organization guidelines (WHO, 2007, 2016), and disposal with appropriate karakia (Māori prayer) will be offered to participant if needed.

### SDB and daytime sleepiness questionnaires

SDB associated symptoms will be assessed using the Pediatric Sleep Questionnaire and Sleep Related Breathing Disorder (SRBD) subscale (PSQ-SRBD; Chervin et al., 2000). This 22-question tool will be filled in by parents and includes questions about history of breathing difficulties during sleep, snoring quality and frequency, daytime signs of sleepiness, Inattention and hyperactivity, in addition to few other questions about morning headache, obesity, enuresis, and delayed growth. The optimal SRBD scale cut-off to indicate presence of SDB would be 0.33 (i.e., ≥33% of the 22 question-items answered positively; Chervin et al., 2000).

Daytime sleepiness and related behavioral disturbances will be assessed using the Epworth Sleepiness Scale (ESS; Manni et al., 1999). ESS is a self-administered questionnaire with 8 questions; it provides a measure of a person's general level of daytime sleepiness, or their average sleep propensity in daily life. It has become the standard method for making this assessment. The ESS asks people to rate, on a 4-point scale (0–3), their usual chances of dozing off or falling asleep in eight different situations or activities that most people engage in as part of their daily lives. The total ESS score is the sum of 8 item-scores and can range between 0 and 24. The higher the score, the higher the person's level of daytime sleepiness (Johns, 1991, 1993; Manni et al., 1999). To make this questionnaire suitable for children, the question about alcohol will be removed and the questionnaire will be completed by parents/caregivers. PSQ-SRBD and ESS questionnaires will be administered four times during the study (Figure 4).

### Quality of life

The OSA-18 instrument is a quality-of-life questionnaire focusing on pediatric sleep disordered breathing. It has been used as a screening tool for pediatric OSA (Franco et al., 2000). This questionnaire consists of 18 questions grouped into five subscales: sleep disturbance, physical symptoms, emotional distress, daytime function, and caregiver concerns. The 18 survey items are scored using a 7-point scale, where the parent/caregiver is asked to report how often during the previous 2–3 weeks their child has had specific symptoms using the following response scale: (1) none of the time, (2) hardly any of the time, (3) a little of the time, (4) some of the time, (5) a good bit of the time, (6) most of the time, and (7) all of the time. The total symptom score (TSS) may vary from 18–126 points. A TSS at or above 60 is considered abnormal and is associated with SDB. Scores of 60–80 suggest a moderate impact on the disease-specific quality of life and a score above 80 suggests a large impact (Franco et al., 2000).

OSA-18 questionnaire will be administered four times and parents/caregivers will rate the frequency of symptoms before and at the end of each treatment period (Figure 4).

### Neurobehavioral assessment

Behavior will be assessed using the Behavior Assessment System for Children: second edition (BASC-2). The BASC-2 tool has been widely used in studying behavioral differences in pediatric SDB patients (Reynolds and Kamphaus, 2004).

Parent Rating Scales (PRS) are used to measure both adaptive and maladaptive behaviors in the community and home setting. Parents or caregivers can complete forms at three age levels—preschool (ages 2–5), child (ages 6–11), and adolescent (ages 12–21). The PRS contains 134–160 items and uses a four-choice response format. Clinical and adaptive scales include the following domains: activities of daily living, adaptability, aggression, anxiety, attention problems, atypicality, conduct problems, depression, functional communication, hyperactivity, leadership, social skills, somatization, and withdrawal.

It takes a parent ~10–20 min to complete this form and requires approximately a fourth-grade reading level for completion. Level two questionnaire (ages 6–11) contains 160 items; but the brief questionnaire of this scale consists of 30 items and will be used in our study. This questionnaire will be administered before and at the end of each treatment period (Figure 4).

### Parent-report of nocturnal enuresis

As about 24% of children with sleep apnea have nocturnal enuresis (Kovacevic et al., 2013), urine incontinence will be assessed during the study by parents/caregivers using diaries in both treatment periods to report frequency of enuresis.

### Sleep study scoring

A certified sleep technologist will manually score all PSG data and AHI according to the AASM criteria (Berry et al., 2012). PSG scoring will be performed using a special software RemLogic-E version 3.4 (Embla system, Natus Europe, Planegg, Germany). The scorer will be blinded to the patients' information and to any indication of the treatment period and appliance used, moreover the technologist will have no contact with the participants through the study progress. To test reliability of scoring PSG data, ten PSG recordings will be chosen randomly and re-scored by the same sleep technician to detect intra-rater reliability.

### Statistics

Data will be analyzed by SPSS (SPSS, IBM Corp. Version 22.0. Armonk, NY, USA) to perform descriptive statistics, and a mixed model analysis, and Bonferroni corrected *post-hoc* tests as appropriate. The significance level will be set at *P* = 0.05.

## Discussion and anticipated results

Early treatment of SDB in children is vital to prevent significant health issues that may develop later. Therefore, SDB may have a significant impact on the wellbeing of many children. With a significant number of cases having persistent OSA after adenotonsillectomy or failure to tolerate CPAP treatment, it is worth exploring alternative options to the current advocated treatment modalities. The MAS intervention may be a valuable and effective alternative for patients who have known craniofacial risk factors of SDB, are on long waiting lists for adenotonsillectomy, are not suitable candidates for adenotonsillectomy, or are not able to tolerate or failed either first-line treatments, such as adenotonsillectomy or CPAP (Huynh et al., 2015).

MAS may be particularly suitable for children with SDB, because it is relatively well tolerated and can easily titrated to obtain a incremental advancement of the lower jaw (DeVincenzo and Winn, 1989). MAS can also expand the upper arch (Clark, 1988), is suitable for both mixed and permanent dentitions (Clark, 1988) and has a low failure rate (Morris et al., 1998). The therapeutic rationale is that the majority of orthodontic anomalies (except class III-protruded mandible), benefit from mandibular advancement capable of enlarging the retrolingual space and at the same time promotes lingual advancement (Villa et al., 2002).

Although there is increasing evidence regarding the efficacy of MAS in adults with SDB, there is a paucity of information about their efficacy in children (Carvalho et al., 2007; Chen and Lowe, 2013; Huynh et al., 2015; Nazarali et al., 2015). The few studies carried out to date suffer from methodological flaws including: heterogeneous samples; lack of randomization; limited power to detect a clinically relevant effect; and lack of an adequate control conditions such as the use of a placebo-like appliance. A Cochrane review (Carvalho et al., 2007) has concluded that there is not enough evidence to support the efficacy of oral appliances and functional orthopedic appliances for the treatment of OSA in children. This conclusion was also reached by two recent systematic reviews and a meta-analysis which confirmed that the current evidence is limited, but suggests possible short-term effectiveness for treating pediatric OSA (Huynh et al., 2015; Nazarali et al., 2015). Therefore, it is currently difficult to draw a conclusion regarding the possible efficacy of mandibular advancement appliances in children affected with SDB (Villa et al., 2002; Cozza et al., 2004; Carvalho et al., 2007; Zhang et al., 2013), and there is a strong need to carry out well-designed RCTs.

Cross over RCT design has been widely used in studying efficacy of oral appliances with OSA patients (Chen and Lowe, 2013). Potential advantages of the crossover design that will be used in current study are its robustness and simplicity of application. Despite these advantages, the current study protocol will have some limitations as it depends on short-term evaluation (3 weeks for each treatment period), and the study will rely on subjective assessment of several secondary outcomes by using questionnaires in evaluating these measures by parents/caregivers. In addition to the possibility of having limited power regarding the secondary outcomes because the sample size estimation was assessed only relying on the main outcome (AHI). However, the study will serve to provide descriptors of these, useful for planning further research in the field.

Randomized clinical trials represent the gold standard for the evaluation of therapeutic effectiveness (Mills et al., 2007) therefore, unlike the previous studies, it is expected the current study protocol will provide new robust and reliable information about the possible efficacy of MAS for the management of SDB in children. The crossover design will allow each participant to act as his/her own control, thus increasing the power of the study. This study will also systematically address efficacy of MAS on other important health outcomes related to SDB, such as quality of life, neurobehavioral functioning, and growth hormone changes; outcomes that have not been widely addressed in previous intervention studies investigating pediatric snoring or OSA (Huynh et al., 2015)

## Author contributions

GI: is the main researcher on this project and studying as a full-time Ph.D. student at the University of Otago. Assoc. Prof. BG: is a co-supervisor to this project. Dr. CR: is a co-supervisor to this project. Prof. MF: is the Principal investigator and main supervisor. All authors contributed to the study protocol and writing of this paper.

## Funding

The study is funded by the New Zealand Association of Orthodontists, the New Zealand Ministry of Health and Oral Health, and Cure Kids [3560].

### Conflict of interest statement

The authors declare that the research was conducted in the absence of any commercial or financial relationships that could be construed as a potential conflict of interest.
